# Rapid Cycle Deliberate Practice (RCDP) as a Method to Improve Airway Management Skills – A Randomized Controlled Simulation Study

**DOI:** 10.7759/cureus.5546

**Published:** 2019-09-01

**Authors:** Isabel T Gross, Dennrik G Abrahan, Ambuj Kumar, Julia Noether, Nicole A Shilkofski, Paula Pell, Laleh Bahar-Posey

**Affiliations:** 1 Pediatrics, Yale University School of Medicine, New Haven, USA; 2 Pediatrics, Cleveland Clinic, Cleveland, USA; 3 Internal Medicine, University of South Florida College of Medicine, Tampa, USA; 4 Pediatric Critical Care Medicine, Johns Hopkins All Children's Hospital, Saint Petersburg, USA; 5 Pediatrics, Johns Hopkins University School of Medicine, Baltimore, USA; 6 Pediatric Emergency Medicine, Johns Hopkins All Children's Hospital, Saint Petersburg, USA

**Keywords:** simulation, rapid cycle deliberate practice, procedural training

## Abstract

Background: Paediatric intubations are a relatively rare but critical procedure that requires adequate practice to achieve skillful performance. Simulation is a method to teach intubation skills in a safe environment. Rapid Cycle Deliberate Practice (RCDP), as a method of simulation debriefing, has been shown to improve pediatric resident resuscitation skills. It has not been demonstrated if RCDP can be effectively used in procedural skills training. The objective of this study was to determine if RCDP with feedback in real-time, as well as an opportunity to repeat the action, is superior to a simulation where no feedback is provided during the simulation and is instead provided after the simulation.

Materials and Methods: This was a randomized controlled single-blinded study. All participants were videotaped during a simulated pre-assessment intubation, then received either the intervention (RCDP) or the control teaching (feedback after the simulation), followed by a post-assessment intubation. These videos were scored by two independent raters on an intubation checklist. The primary outcome was the change in score. The secondary outcome was intubation success.

Results: Thirty-five students met the inclusion criteria. The RCDP group achieved a significantly higher score improvement in the preparation and post-procedure care categories. The overall score change in the RCDP group was significantly higher than in the control group, with a mean difference of -11.86 (CI -15.57 to -8.15, p<0.00001), but there was no significant improvement in intubation success.

Conclusion: Our study suggests that RCDP is an effective method to teach the procedural skill of intubation with an emphasis on procedural choreography. RCDP could be an appropriate method for debriefing learners in procedural skills training in this population.

## Introduction

Pediatric intubation, compared to adult intubation, is a relatively rare procedure that requires many hours of practice to achieve skillful performance. However, clinical opportunities for practicing these skills are limited. It is challenging to provide trainees with appropriate opportunities to practice their airway management skills [1]. Pediatric intubations have a low first-attempt success rate, and methods to improve procedural training for intubations need to be identified [2]. Simulation is an educational strategy that can help providers practice their skills in a safe environment that mimics the fidelity of the clinical environment. Intubation is not considered a required procedure by the Accreditation Council for Graduate Medical Education (ACGME) for pediatric residents during their residency training. Nevertheless, many pediatricians will practice in rural or limited-resource areas where they may be the only provider able to secure an airway and intubate a pediatric patient. Various training models for intubation have been used in the past, but we still have not identified a paradigm to reliably and validly improve pediatric intubation skills acquisition [3]. Reliable training models need to be identified in order to optimize instructional simulation designs to achieve learning goals.

Debriefing is a vital component of simulation-based medical education. There are various debriefing techniques described in the literature with no clear evidence of the superiority of one method over another [4]. Rapid Cycle Deliberate Practice (RCDP) as a method of within-event debriefing has been shown to improve pediatric resident resuscitation [5]. It has also been shown to be an effective teaching method for neonatal resuscitation training [6]. When using RCDP, we allow learners multiple opportunities to “do it right” while giving them direct feedback until they master the skill [5]. Micro-debriefs with reflection-in-action “pause and discuss/rewind” as a form of post-event debriefing may be differentiated from debriefing at the end of a simulation without interruption [7]. Although RCDP is a new teaching method in simulation-based training, programs have started incorporating it into continuing professional development [8]. In addition, it is potentially beneficial for team performance and human factors improvement within high fidelity simulations [9]. To date, RCDP has been shown to be primarily effective for pediatric and neonatal resuscitation training [10]. To our knowledge, RCDP has not been demonstrated to be effective for procedural skills task training. The objective of this study was to determine if RCDP is superior to feedback after the simulation during procedural skills training in pediatric intubations for novice trainees.

## Materials and methods

Trial design

This was a randomized controlled simulation-based study. The allocation ratio was 1:1, and we applied a superiority framework assessing if the RCDP group performs superior to the group that received feedback after the simulation. The Institutional Review Board of the University of South Florida approved this study.

Blinding

The study was single-blinded. The facilitators and participants were aware of the group assignment. The raters were blinded to the group assignment. The data analyst was only made aware of the groups as per the research number and not the actual assignment.

Power calculation

Eighty-six subjects (43 in each arm) were required to have a 90% chance of detecting, as significant at the 5% level, an increase in the primary outcome measure of intubation skill from 3.4 in the control group to 4.8 in the RCDP group. However, the enrollment stopped after the first year as the logistical support to continue the study was not available after enrollment of 35 subjects. A post-hoc power calculation with 35 subjects and an observed mean difference of 12.33 (95% CI 6.2 to 18.5) had 95% power to detect this difference.

Randomization

Sequence Generation

We generated a stratified block randomization sequence using a computer program with random block sizes.

Allocation Concealment

The group assignment was performed by a designated research coordinator not involved in the pre-assessment, post-assessment, or intervention. All participants were assigned a number that was marked on a badge-sticker. The knowledge of number coding was available to the statistician and research coordinator only.

Study setting and participants

The study site was the Centre for Advanced Medical Learning and Simulation (CAMLS) at the University of South Florida in Tampa, Florida. Our study population consisted of 33 fourth-year medical students and two pharmacy students at the University of South Florida. Amongst the student cohort, there were pharmacy students interested in this teaching opportunity as well. The students were volunteers rotating through different teaching stations offered during an educational event and included students who had matched into any specialty.

Intervention

We recruited participants in a scheduled residency preparation day by email, explaining the purpose of the study as examining ways to give feedback on intubation skills on a simulation task trainer. Volunteers filled out a questionnaire assessing demographic data [age, gender, intended specialty, and experience with previous intubations (none, 1-5, 6-10, 11-20, >20)]. We used a simulation task trainer for airway management (SimBaby head; Laerdal Medical, Stavanger, Norway) during all components of the study. Participants were shown a standardized video of an intubation where an expert performed all required steps to intubate a newborn on a Laerdal SimBaby [11]. After this, all participants were individually oriented to the simulation task trainer, and all available equipment was shown to them. They then attempted intubation, which was videotaped for assessment by two investigators independently using a checklist [12]. All participants were exposed to the same mannequin and given the following vignette: “You are called to the bedside of this 9-month-old, 9-kilogram patient and asked to secure the airway of this patient”. Each participant subsequently initiated the required steps to intubate the mannequin. After this baseline intubation had been videotaped, the participants were randomized to two different groups using either feedback after the simulation (control group) or RCDP style debriefing (intervention group) and asked to intubate the mannequin a second time. This second attempt was also video captured.

Intervention group (RCDP)

Participants randomized to the RCDP group were stopped immediately mid-procedure if they failed to perform a specific task that was deemed necessary for safe intubation. If the student performed each set of skills correctly, they were allowed to proceed with each task uninterrupted. Each time the student was stopped, she or he received direct feedback and was asked to start the procedure over again from the beginning. The feedback given amongst the different participants was standardized through a facilitator training session prior to the initiation of the study. The checklist served as a guideline for feedback. The facilitators had the same checklist on hand that would later be used during the video review to grade the students. Students were asked to prepare for their intubation, announce “I am ready to intubate” when they felt ready to do so, verbalize when they visualized the vocal cords, and perform the intubation. Each simulation room was set up in the same way with the same equipment available. Tube sizes available were cuffed 2.5, 3.5, 4.5, and size 3.5 was considered the appropriate endotracheal tube (ETT) size for stated mannequin age. We offered Miller 0, Miller 1, Mac 2 blades and considered Miller 1 the correct choice for stated mannequin age. We identified three stopping points. At each stopping point, the student was interrupted, provided with direct feedback, and asked to go back and repeat the procedure until the stopping point. Once performed correctly, the student would proceed until stopped at the next stopping point. The first stopping point was after the “preparation phase” was completed marked by the student announcing, “I am ready to intubate.” The second stopping point was after the student had passed the tube through the vocal cords, marking the end of the “procedure phase”. The third stopping point was after completing the “post-procedure phase”.

Once the participants successfully completed the intubation, no additional debrief was provided. Immediately following the completion of the simulated training, the participant was asked to perform an intubation that was videotaped and rated using the same checklist used in real-time during the initial intubation assessment. This marked the end of the study protocol, followed by a period of time to address any unanswered questions.

Control group (feedback after the simulation)

Participants randomized to the control group received a debrief after the simulation had ended that included the same learning objectives that were used in the RCDP group. As in the RCDP group, the facilitators had the same checklist on hand that would later be used during the video review to grade the students. The debrief was not scripted, but the structure followed the advocacy inquiry framework [12]. Facilitators demonstrated haptic skills on the simulator with the participants. Following the debrief, the student was asked to perform an intubation that was videotaped and rated with the same checklist used during the initial intubation assessment. This marked the end of the study protocol for this group, followed by a period of time to address any unanswered questions and any major problems encountered during post-intervention intubation. Thereafter, this group was equally offered a separate room to independently practice their intubation skills after their participation in our study. Each participant was assigned a total of 15-minute teaching time in both the intervention and in the control arm. The facilitators were informed about the random group assignment and served as facilitators for both arms.

Measurement tool

The checklist used for this study is reported in Johnston et al. 2019 [13]. This checklist was reported to have high validity evidence when used for simulated neonatal intubations. It contains 22 items, and each item is weighted equally. Each item is graded with zero points (not done or done incorrectly), one point (done with prompt or done partially), or two points (done independently or done correctly with no prompts). For this study, we used 19 of the 22 items on the checklist, not assessing the items “verbalizes the indications for the procedure, verbalizes the risks and/or contraindications for the procedure, and equipment check”.

Primary and secondary outcomes

The primary outcome was the change in score on the intubation checklist in the post-simulation intubation compared to the initial assessment intubation (i.e., change from baseline). The secondary outcome was the success rate of the intubation (endotracheal position successfully passed through the vocal cords allowing ventilation).

Statistical methods

All data were entered online using Qualtrics software into an encrypted database which was password protected. The servers are protected by high-end firewall systems and scans are performed regularly to ensure that any vulnerabilities are quickly found and patched. Summary descriptive statistics were constructed using frequencies and proportions for categorical data elements and means and medians along with ranges for continuous variables. An independent sample t-test and Fisher's exact test were used to compare the difference in intubation skills score and success rate of intubation, respectively. A p-value of less than 0.05 was considered to be statistically significant. Data for continuous outcomes were summarized as mean difference and binary outcomes as relative risk along with 95% confidence intervals (CI). All analyses were performed as per the intention-to-treat (ITT) principle. There were two raters for each scenario. An inter-rater reliability analysis using the Kappa statistic was performed to determine consistency among raters [14].

## Results

Participant flow and baseline characteristics

**Figure 1:**
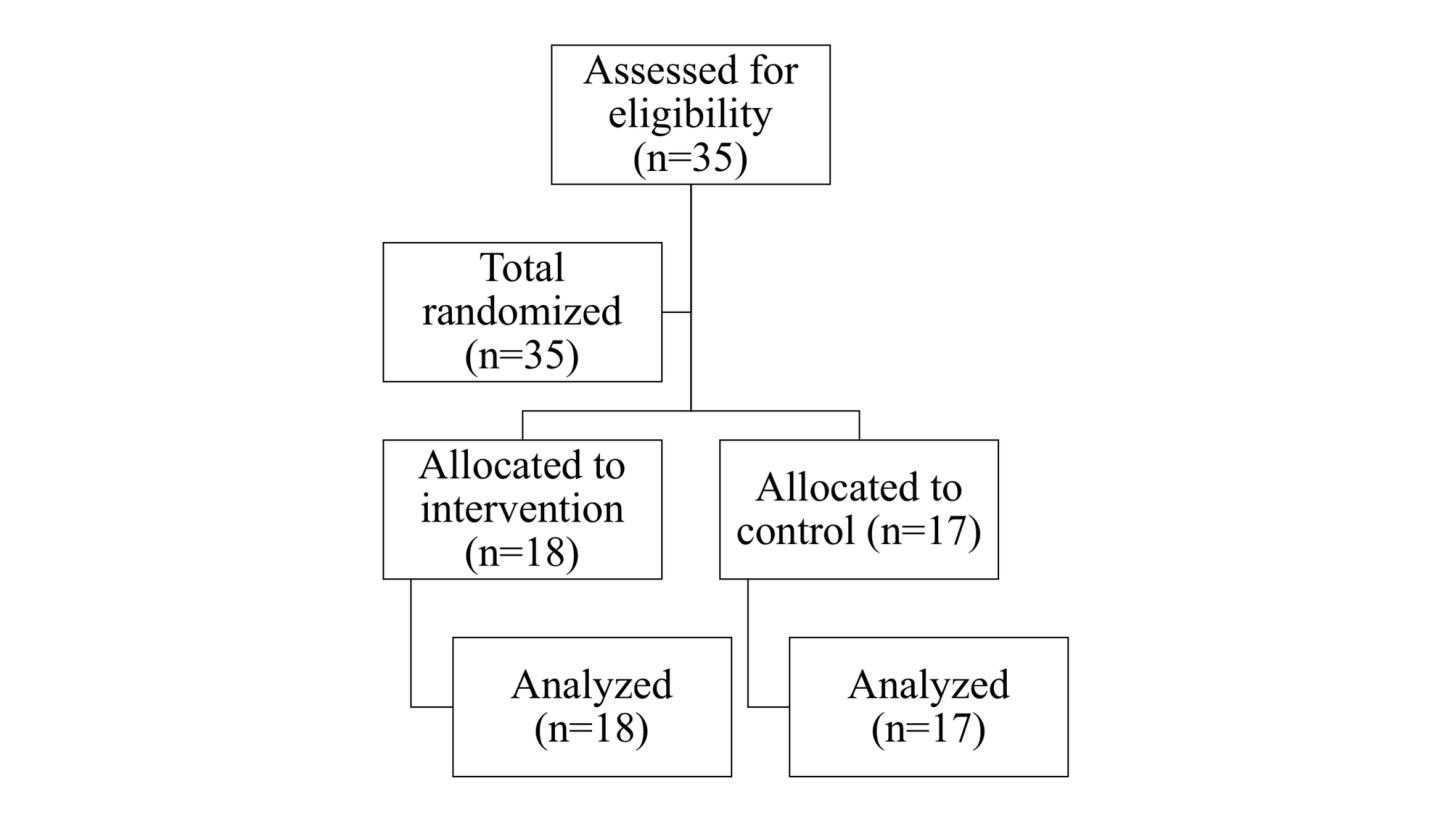
Participant flow diagram

Thirty-five participants were eligible and randomized to the RCDP or the control arm (Figure 1). There was no statistical difference between these two groups in their demographic characteristics (Table 1). The mean age of participants in the RCDP arm and control arm was 27 years (±2.6). There was no loss of follow up.

**Table 1:** Demographics and participant characteristics

	Control Group n=17 (%)	Intervention Group n=18 (%)
Gender		
Female	9 (52.9)	9 (50)
Male	8 (47)	9 (50)
Prior intubations		
0	15 (88.23)	18 (100)
1-5	1 (5.9)	0 (0)
6-10	1 (5.9)	0 (0)
>10	0 (0)	0 (0)
What specialty did you match for?		
Pediatrics	7 (41.2)	3 (16.7)
Family Medicine	1 (5.3)	1 (5.6)
Medicine/Pediatrics	1 (5.3)	3 (16.7)
Surgery	2 (10.5)	2 (11.1)
Emergency Medicine	0 (0)	2 (11.1)
Clinical Pharmacy	0 (0)	2 (11.1)
Radiology	1 (5.3)	2 (11.1)
Anesthesiology	1 (5.3)	1 (5.6)
Obstetrics/Gynecology	1 (5.3)	1 (5.6)
Internal Medicine	0 (0)	1 (5.6)

Outcomes

Primary Outcome

**Figure 2:**
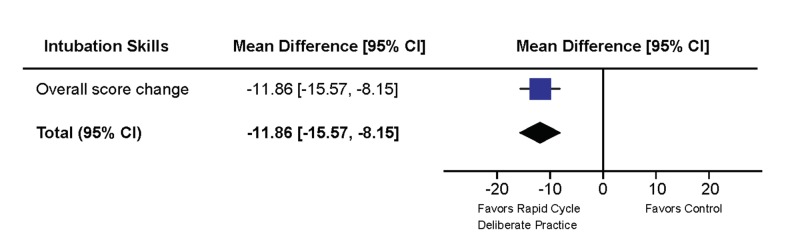
Change in the over-all intubation checklist score. Mean differences with 95% CI are displayed. Values to the left of the middle bar represent results favoring RCDP, values to the right of the middle bar represent results favoring the control. The width of the diamond represents the spread of data, the size of the diamond represents the effect size.

The primary outcome was the improvement in intubation choreography as measured by the score on the intubation checklist in the post-simulation intubation compared to the initial assessment intubation. The overall score change in the RCDP group was significantly higher than in the control group with a mean difference of -11.86 (CI -15.57 to -8.15, p<0.00001) (Figure 2)

**Figure 3:**
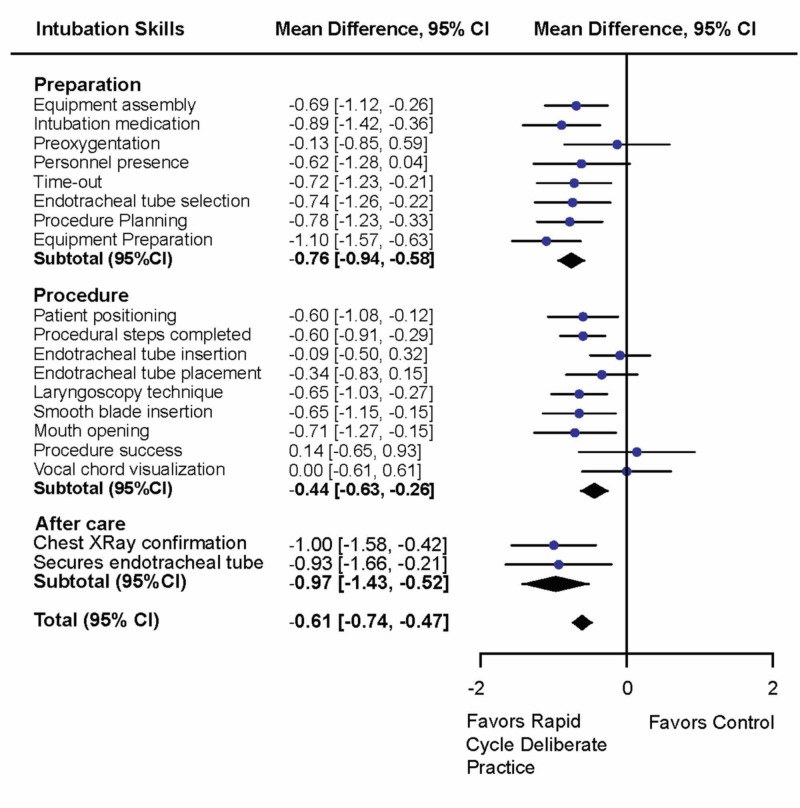
Intubation checklist score divided by subgroups: preparation, procedure, after-care, total score within each subgroup Mean differences with 95% CI are displayed. Values to the left of the middle bar represent results favoring RCDP, values to the right of the middle bar represent results favoring the control. The width of the diamond represents the spread of data, the size of the diamond represents the effect size.

Participants receiving RCDP training achieved a significantly higher score improvement than participants that received intubation training with post-scenario feedback. When analyzing the items on the intubation checklist, we divided the necessary steps into categories consisting of preparation, procedure and post-procedure care (Figure 3).

Secondary Outcome

The secondary outcome was the success rate of intubation (defined as endotracheal positioning through the vocal cords as measured by visible mannequin lung insufflation). There was no significant difference in the mean difference between the two groups for endotracheal tube placement success (mean difference 0.14, CI -0.69 to 0.93) (Figure 3).

Inter-rater agreement between reviewers

The interrater reliability was found to be between Kappa = 0.480 (p<0.001) and Kappa = 0.784 (p<0.001) when assessing interrater reliability for all checklist items. This measurement of agreement is considered moderate agreement (moderate agreement = 0.41 - 0.60) to substantial agreement (substantial agreement = 0.61 - 0.80) [14].

## Discussion

This study is the first to assess if RCDP is superior to a methodology where feedback is provided after the simulation to teach procedural skills. The primary outcome of overall score improvement on the simulation checklist significantly favored the RCDP group over the control group, suggesting that RCDP is an effective simulation debriefing method for procedural skills training. Even though endotracheal intubation is a composite procedural skill, a comprehensive choreography needs to take place to ensure the success of the procedure. This choreography was more accurately performed in the group that received RCDP. RCDP has been shown to be effective when teaching resuscitation skills [5]. Thus, highly choreographed or algorithm-based skills might be best learned and taught using RCDP methodology. While the overall scores significantly improved, we observed that the rate of successful endotracheal tube placement did not significantly differ between the two groups. From an educational perspective, the intubation choreography is likely more important than the success of placing the endotracheal tube in the correct position, especially in the novice learner. In the early stages of training, it is imperative to build proper technique when intubating with the focus on the procedure, and not just the outcome of successful intubation. If the procedure is not performed in a well-choreographed manner, successful intubation may promote poor performance habits that would later be dangerous for the learner. In our study, we did not show a statistically significant difference in students’ ability to place the endotracheal tube in the correct position, raising the question if RCDP is superior for the haptic aspect of procedural training. Less sophisticated haptic components such as equipment assembly, positioning, use of the laryngoscope, blade insertion, and mouth opening technique were significantly improved in the RCDP group. We acknowledge that one single intubation training session will unlikely be sufficient to train students to achieve mastery in the complex skill of endotracheal intubation, but mastery could be achieved for other haptic procedural skills.

Limitations

This study was a single-center study, including a limited number of participants, which may limit external validity and generalizability. This could be addressed in future studies by using several different learner levels, as well as extending it to a multi-center study. We were assessing students that had limited or no experience with endotracheal intubations, and it was not expected that mastery of this skill could be achieved during a single educational intervention. Enrolling more experienced learners and repeat simulations over time could potentially help further explore the utility of RCDP in procedural skills training. An additional limitation is that we did not assess skill retention or decay, given that we performed our study during one single educational event.

Future directions

The results of this study will serve as a foundation to use RCDP for procedural skills training. It will be crucial to assess if performance improvements seen in this simulation-based intervention are transferable to actual clinical environments in order to demonstrate an impact on patient outcome. Following students over time to assess skill retention vs. decay, and repeating the teaching intervention at defined time intervals will be necessary to draw further conclusions regarding skills acquisition using RCDP compared to methods providing feedback after the simulation.

## Conclusions

Our study suggests that RCDP is an effective method to teach the procedural skill of intubation with an emphasis on procedural choreography. The overall score improvement on the simulation checklist significantly favored the RCDP group over the control group, suggesting that RCDP is an effective simulation debriefing method for procedural skills training. However, a longitudinal curriculum and a larger multi-center approach involving multiple different procedures will be needed to show significant improvement in the mastery of complex procedural skills using RCDP.
